# Beyond conventional microscopy: Observing kidney tissues by means of fourier ptychography

**DOI:** 10.3389/fphys.2023.1120099

**Published:** 2023-02-13

**Authors:** Marika Valentino, Vittorio Bianco, Lisa Miccio, Pasquale Memmolo, Valentina Brancato, Paolo Libretti, Marcello Gambacorta, Marco Salvatore, Pietro Ferraro

**Affiliations:** ^1^ National Research Council (CNR) of Italy, Institute of Applied Sciences and Intelligent Systems (ISASI), Pozzuoli, Italy; ^2^ Department of Electric and Information Technologies Engineering, University of Naples “Federico II”, Naples, Italy; ^3^ IRCCS SYNLAB SDN, Naples, Italy

**Keywords:** renal tissue morphology, fourier ptychographic microscopy, unstained morphology, quantitative phase imaging, kidney physiology

## Abstract

Kidney microscopy is a mainstay in studying the morphological structure, physiology and pathology of kidney tissues, as histology provides important results for a reliable diagnosis. A microscopy modality providing at same time high-resolution images and a wide field of view could be very useful for analyzing the whole architecture and the functioning of the renal tissue. Recently, Fourier Ptychography (FP) has been proofed to yield images of biology samples such as tissues and *in vitro* cells while providing high resolution and large field of view, thus making it a unique and attractive opportunity for histopathology. Moreover, FP offers tissue imaging with high contrast assuring visualization of small desirable features, although with a stain-free mode that avoids any chemical process in histopathology. Here we report an experimental measuring campaign for creating the first comprehensive and extensive collection of images of kidney tissues captured by this FP microscope. We show that FP microscopy unlocks a new opportunity for the physicians to observe and judge renal tissue slides through the novel FP quantitative phase-contrast microscopy. Phase-contrast images of kidney tissue are analyzed by comparing them with the corresponding renal images taken under a conventional bright-field microscope both for stained and unstained tissue samples of different thicknesses. In depth discussion on the advantages and limitations of this new stain-free microscopy modality is reported, showing its usefulness over the classical light microscopy and opening a potential route for using FP in clinical practice for histopathology of kidney.

## 1 Introduction

Light microscopy plays an important role in studying kidney tissue and in analyzing the renal complex morphology and functioning, especially in evaluating pathological assumptions. Hence, the improvement of microscopical techniques is an essential point in this process. Electron microscopy (Howell and Herrera Guillermo, 2021), confocal microscopy (Unnersjö-Jess D. et al., 2018), fluorescence microscopy (Shroff, U. N. et al., 2019), immunohistochemistry (Weening and Jennette, 2012), X-ray microscopy (Rick R. et al., 1986), and the recent more sophisticated techniques, such as multiphoton microscopy (Schuh C.D. et al., 2016), expansion microscopy (Chozinski, T.J. et al., 2018), super resolution microscopy (Unnersjö-Jess D. et al., 2016) and cryo-electron microscopy (Su, Q. et al., 2018) add different information to light microscopy, offering distinct sights of the same specimen (Angelotti, M. L. et al. 2021), useful for clinicians to better understand kidney physiology and pathology. However, due to the inherent limitations of any classical optical system, there is still a lack of a microscope capable of providing high-resolution object details over a large field of view. In fact, microscope objectives having limited numerical apertures (NAs) and low magnification allow to image a large area of interest to observe the macroscopic morphology and behavior of biological samples (e.g., cell spreading processes or the entire tissue architectures), but at the cost of low spatial resolution, which results in the loss of the smallest details. Conversely, high spatial resolution is achievable by selecting higher NA objectives, but only over a limited field of view. Sample scanning approaches should be adopted in this case to cover the entire region of interest, which is a time-consuming operation and computationally demanding.

Here we show, for the first time at the best of our knowledge, the use of Fourier Ptychography (FP) (Zheng et al., 2013) for the inspection of kidney tissue slides with optical performance that are superior in respect to the classical light microscopy instruments commonly used in histopathology. Moreover, such FP imaging method has the significant advantage of avoiding any tissue staining, since the optical readout is the phase delay the specimen introduces on the incoming light beam, which serves as a contrast mechanism to visualize transparent samples. FP is recognized as a low-cost quantitative phase method (Popescu, 2011; Ou X. et al., 2013; Park et al., 2018), providing both large field of view and high-resolution images (Zheng et al., 2013). The FP system uses a microscope objective with low NA to attain vast sample areas and multiple angled light-sources (generally a LEDs matrix) that sequentially illuminate the object to reach high-resolution exploiting a synthetic aperture principle. Combining the spectral contribution of each tilted illumination wave, a bigger synthetic NA is generated, and fine details can be recovered. Hence, during the acquisition process, raw images are captured and given as input to phase retrieval algorithms for reconstructing the object complex amplitude (Yeh et al., 2015). Accordingly, amplitude and phase contrast images are made available after an iterative estimation process. Since its first introduction in 2013, FP technology has rapidly grown over the years and different configurations have been proposed for biology studies. Tian et al. studied *in vitro* cell cultures, observing in time-lapse experiments the growth and the confluence of live cells (Tian et al., 2015); Williams et al. demonstrated how FP can detect tumor cells (Williams Anthoy et al., 2014). Chung et al. showed the feasibility to count white blood cells in the blood smear acquired by FP (Chung et al., 2015); Wakefield et al. analyzed and counted label-free cells from FP images (Wakefield Devin et al., 2022); Horstmeyer et al. used FP for imaging histological specimens to improve digital pathology with FP quantitative information exploiting the scattering properties of tissue samples (Ou X. et al., 2013); Song et al. proved the birefringence of Tilia stem cells using a polarization-sensitive FP microscope (Song et al., 2019). Pirone et al. used FP in the field of mechanobiology, studying the process of cells adhesion onto functionalized micropatterned substrates (Pirone et al., 2022). Newly, FP has been coupled to a deep learning architecture to obtain real-time reconstructions of tissue slides avoiding fine optical alignments of the setup (Bianco et al., 2022), which is an important step toward the widespread use of this technology by non-experts.

Of note, FP has the peculiarity of obtaining phase contrast imaging of unstained biological samples, without adding external stains (e.g., Hematoxylin and Eosin (H&E)). We demonstrate that such phase images given by FP are capable to preserve all the morphological spatial features of the unstained objects thus avoiding any potential disturbing effect of the used contrast agents (Kim et al., 2016; Tsutsumi, 2022).

To show how kidney structures appear in FP phase-contrast images, we probed stained renal tissue slides to let physicians compare light microscopy images with the FP ones. After highlighting the differences between the two modalities, a comparison between FP-unstained and FP-stained images is illustrated to point out the preserved visibility of the inner architectures of unstained kidneys, such as glomeruli, tubules, and vascular sections.

Moreover, we acquired unstained renal tissue slides with different thicknesses to evaluate the limits of FP method in resolving thicker slides. We demonstrate that FP can resolve 10 µm thick renal tissue slides while preserving three-dimensional information, thus overcoming one of the side effects of conventional light microscopy relying on staining processes, which is intrinsically limited in terms of maximum slide thickness.

It is worth to mention that FP systems could be compacted, automatized and applied in clinical practice (Konda P. C. et al., 2020; Bianco et al., 2021 *;* Bianco et al., 2022), allowing unexperienced operators to easily manage and use them as an aid tool for biological samples investigations and histological diagnostics. In the present work, our FP experimental outcomes on kidney tissues pave the way for the possibility of finding innovative features for more accurate and reliable pathological discriminations.

## 2 Results

An experienced physician selected several regions of interest (ROIs) of kidney tissue slides, relying on conventional microscopic renal tissue images captured by surgical samples of two kidneys and marked with different stains, i.e., H&E, CKpool, AML, FVIIIra, PAS, Blue Tol, and Giemsa (see Section 4.1 for more details). The light microscopy images of stained slides were captured with Philips scanner UFS. The same ROIs have been acquired by a custom FP setup, scanning the renal tissue slides with motorized XY-stages. Once the in-focus corresponding portions were found, FP datasets were collected for our purpose. The thickness of the probed renal tissue slides is 3 μm for the stained renal slides.

In the FP images, the absorption of light changes according to the type of staining applied to the slide, still preserving the details of the inner structures of renal tissues that are normally highlighted by using different stains.

As mentioned above, the readout of the FP system is the optical phase delay introduced by the specimen on the impinging light probe. The FP processing results in a wide field of view, super-resolved image, namely, with 0.5 μm lateral resolution over ∼3.3 mm^2^ area. One of the main characteristics of FP is the capacity to capture images of unstained slides providing a new type of data exhibiting enough contrast for downstream analysis. Therefore, we imaged unstained kidney slides with different thicknesses through FP. In particular, 3 μm slides were stained with H&E, Ckpool, AML, FVIIIra, PAS, Blue Tol, and Giemsa, while unstained slides of the same paraffin blocks were cut at 3, 10, 20, and 30 μm and after FP acquisition were stained with H&E, re-scanned and examined again.

**FIGURE 1:**
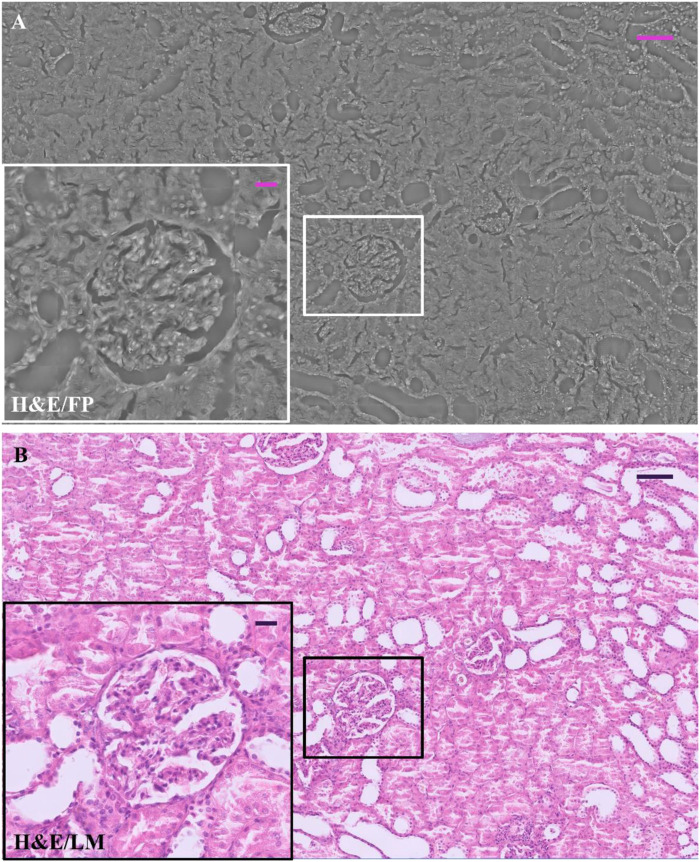
H&E-stained renal tissue: **(A)** Whole renal tissue FP image; the inset (white box) zooms glomerulus captured by FP; **(B)** whole renal tissue LM image; the inset (black box) zooms glomerulus captured by LM. The scale bar in **(A)** and **(B)** is 105 μm, while the scale bar in the respective insets is 25 µm.

In Figure 1, an example of the entire field of view of a renal tissue phase map acquired by FP shows the possibility of studying, over a wide renal tissue portion, the morphological structure as a whole and, at the same time, the microscopic details of glomeruli up to the single cell level. We compare the FP image of the H&E-stained slide, Figure 1A, with its Light Microscopy (LM) counterpart, Figure 1B. Zoomed in details, glomerulus and tubules section is shown in the insets of the Figure 1.

**FIGURE 2:**
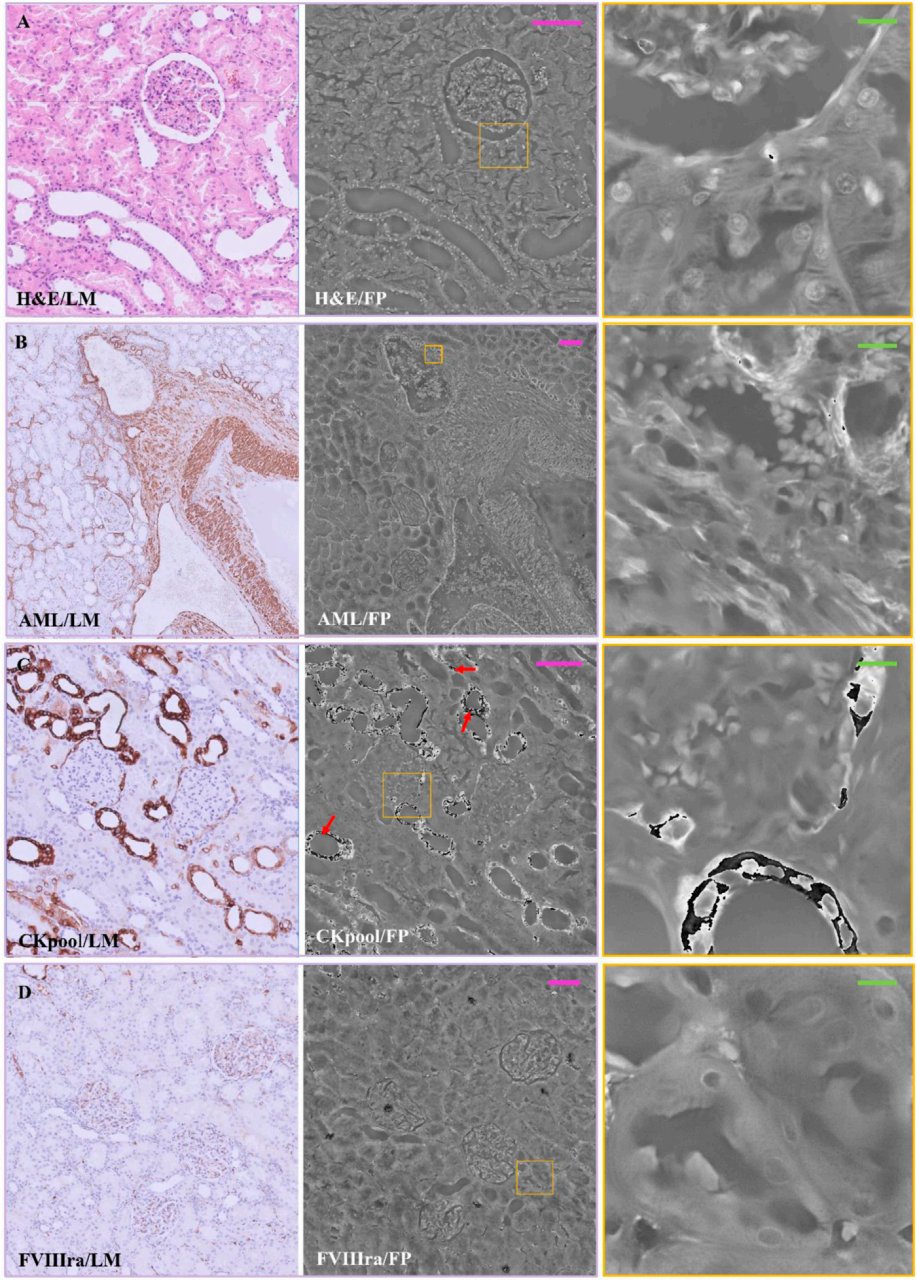
Stained renal tissue slides: Renal tissue portions with 3 µm thickness are stained with various dyes and captured by (left) LM and (center/right) FP. **(A)** H&E; **(B)** AML; **(C)** CKpool; **(D)** FVIIIra. Scale bars: 105 µm. Red arrows in C indicate the phase jumps. On the right, the zoomed patches of the corresponding yellow boxes have the green scale bar of ∼13 µm.

In Figure 2, a comparison between the two imaging modalities is illustrated for H&E, AML, Ckpool, and FVIIIra stains. The images are paired according to the used stain, where the RGB LM image is on the left-side, while the FP phase image is reported on the central/right-side. As expected, the stains highlight different areas of the renal tissue, resulting in different local absorption of light and different local optical phase delays that are encoded in the FP maps. The H&E, in Figure 2A, accumulates in cell nuclei that are shown with good contrast in the LM image.

Besides, a first outcome of the experimental campaign we carried out is the assessment of the nice contrast of the nuclei also in the FP phase image of the H&E-stained slide. The whole renal tissue morphology is clearly inferable while each single nucleus shows local maxima of the phase values, as the corresponding zoomed patch illustrates on the right-side. Conversely, in Figure 2B, the AML stains the smooth muscle fibers, which appears not enough contrasted when imaged in FP modality. Zooming a portion (Figure 2B right), the AML stain coincides with the highest phase values (i.e., white color in the image, which tends towards π value). In Figure 2C, the Ckpool emphasizes renal tubules and Bowman’s capsules, whose values are higher in the FP phase map. However, the strong absorption of the stain provokes phase jumps (Judge and Bryanston-Cross, 1994; Montrésor et al., 2019; Park S. et al., 2021) in the FP map (see the red arrows in Figure 2C, zoomed on the right), which should be corrected by phase unwrapping methods (Hwang W-J et al., 2011).

**FIGURE 3:**
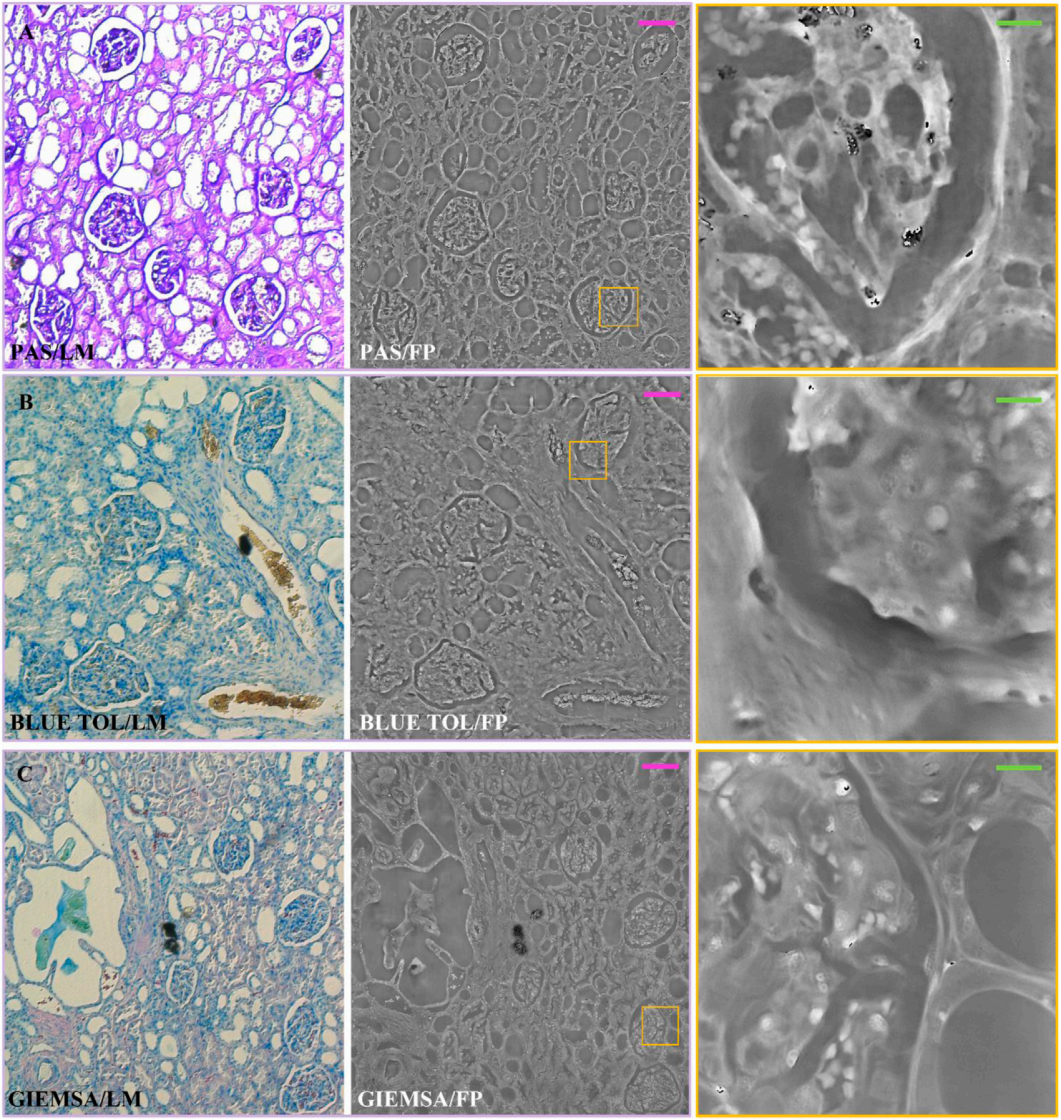
Stained renal tissue slides: Renal tissue portions with 3 µm thickness are stained with various blue dyes and captured by (left) LM and (center/right) FP. **(A)** PAS; **(B)** BLUE TOL; **(C)** GIEMSA. Scale bars: 105 µm. On the right, the zoomed patches of the corresponding yellow boxes have the green scale bar of ∼13 µm.

Finally, in Figure 2D, the FVIIIra dye reaches the vascular endothelium. The corresponding areas result in higher phase contrast in the FP maps, detailed in the zoom on the right-side. In Figure 3, the comparison between the two imaging modalities for PAS, Blue Tol and Giemsa stains is illustrated. PAS stain, Figure 3A, marks carbohydrate macromolecules in the connective tissue, mucus, etc., resulting in higher phase values in correspondence to the edges of the tissue structures in the FP images. The effect of PAS stain is zoomed on the right-side, where its accumulation points generate phase jumps. Blue Tol, Figure 3B, accumulates in nucleic acids generally to identify mast cells and cartilage, but FP image does not show the effect of the stain in a particular phase intensity variation. Instead, GIEMSA stains microorganisms present in tissues, especially the cores in blue and the cytoplasm in pink (Figure 3C). The blue cores are brighter in the FP counterpart, Figure 3C central/right-side, thus highlighting the effect of the GIEMSA staining.

**FIGURE 4:**
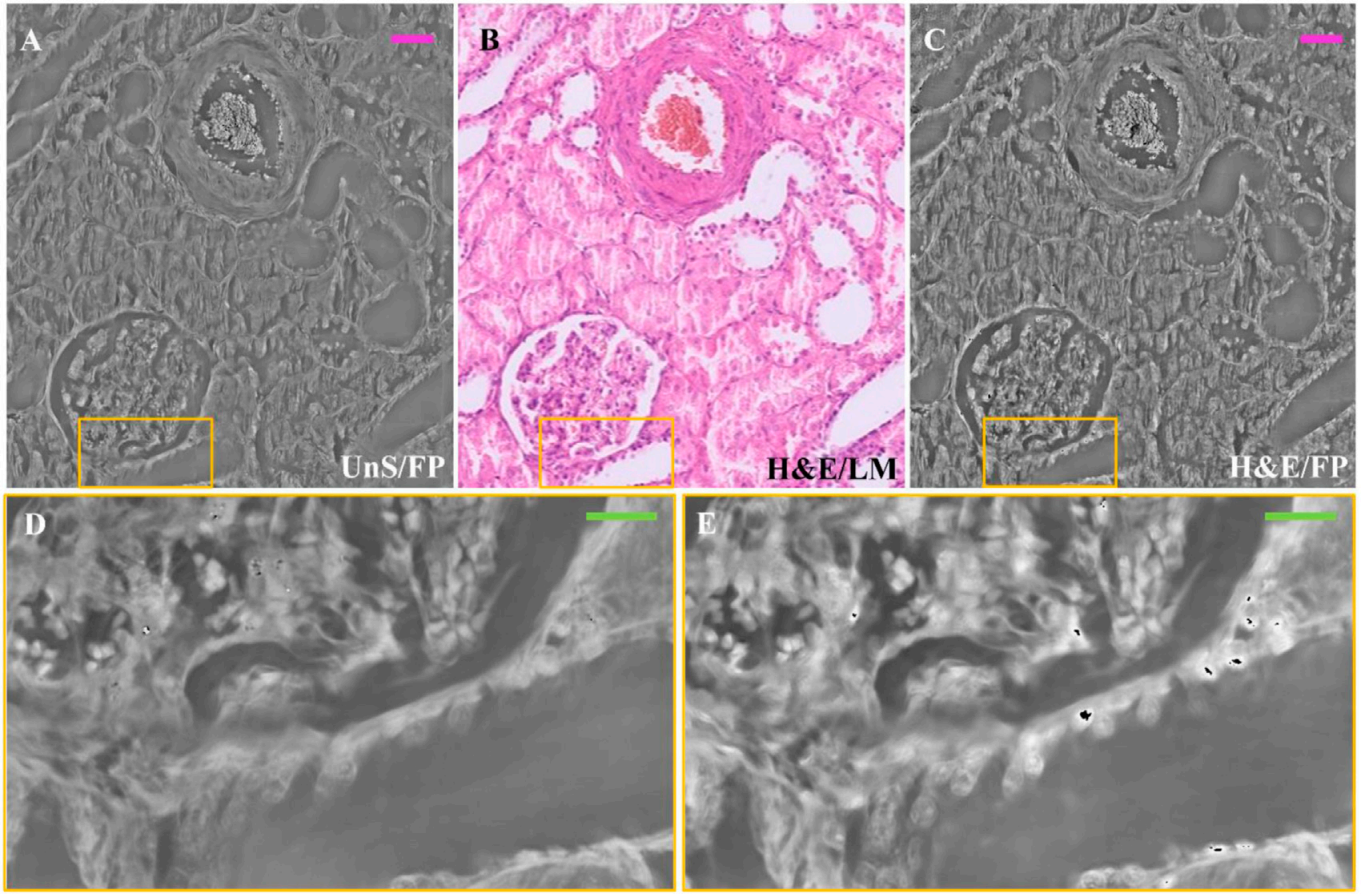
Unstained and H&E-stained images comparison: **(A)** Unstained renal tissue portion captured by FP; **(B)** H&E-stained renal tissue portion captured by LM as gold standard; **(C)** H&E-stained renal tissue portion captured by FP. The scale bar is ∼50 μm; **(D)** zoomed area of the unstained renal tissue of the yellow box in **(A–E)** zoomed area of the H&E-stained renal tissue of the yellow box in **(C)**. The green bar is ∼17.5 µm.

In order to compare the same section of tissue before and after applying the staining process, we captured FP images of a 3 μm thick unstained renal tissue slide, then we performed H&E staining and made LM and FP acquisitions of the same structure. In Figure 4, an example of the same ROI of the H&E-stained renal tissue slide is compared with the unstained one. The selected ROI contains one glomerulus and a vascular section, as the LM gold standard shows (Figure 4B). Noteworthy, the unstained phase contrast image (Figure 4A) highlights the renal tissue components, similarly to its H&E-stained version (Figure 4C). In Figures 4D, E, the zoomed areas corresponding to the yellow boxes in Figures 4A–C are compared to show the contrast enhancement where the H&E stain accumulates. Nevertheless, the high-contrast areas corresponding to the nuclei in the H&E-stained renal tissue slide in Figure 4E are distinguishable as well in the stain-free FP image of Figure 4D.

Noteworthy, although both images in Figures 2, 4B, C correspond to H&E-stained tissue of the same sample, they were obtained from two different sections (stained with H&E at different times). This visual diversity highlights the well-known drawbacks related with H&E-staining process such as the lack of reproducibility and the color variations. Concerning the former, the missing reproducibility is linked with the possible H&E staining inconsistency, with results varying between different laboratories, staining technicians and even between samples within a laboratory. Concerning color variations, H&E staining can produce different shades of color intensity and hue, which can lead to variations in interpretation of results. To mitigate these effects, solutions such as the use of standard operating procedures for staining and stain normalization were proposed by researchers, as variations in the concentration of the solutions can impact the final coloration and affect results (Azevedo Tosta TA et al., 2019; Chlipala E. A. et al., 2021). In our study, the staining procedure was implemented following the same pre-analytical procedure. However, drawbacks related to color variations are intrinsic to the process. In further studies performed, for example, on quantitative data arising from the investigated images (e.g., pathomics) it could be interesting to study the effect of color variations both using normalization approaches or not. Investigations without normalization could be advantageous in terms of generalizability of the approach developed in a clinical context since the dataset investigated could be representative of real-world data acquired in clinical practice.

Results confirm that FP is suitable to image unstained renal tissue slides, allowing to obtain quantitative information without being compromised by stains influence. One possible application could be performing preliminary FP mapping of the slide before applying a stain on it. This type of image would by far outperform in contrast the image obtainable by LM, since any unstained slide typically shows very poor contrast signature and appears almost transparent under LM. Then, the set of morphometric information inferable from the FP phase-contrast map could be used to select better the staining process to apply to the slide for further histological analysis, which could be better tailored to the specificities of the slide under test.

**FIGURE 5:**
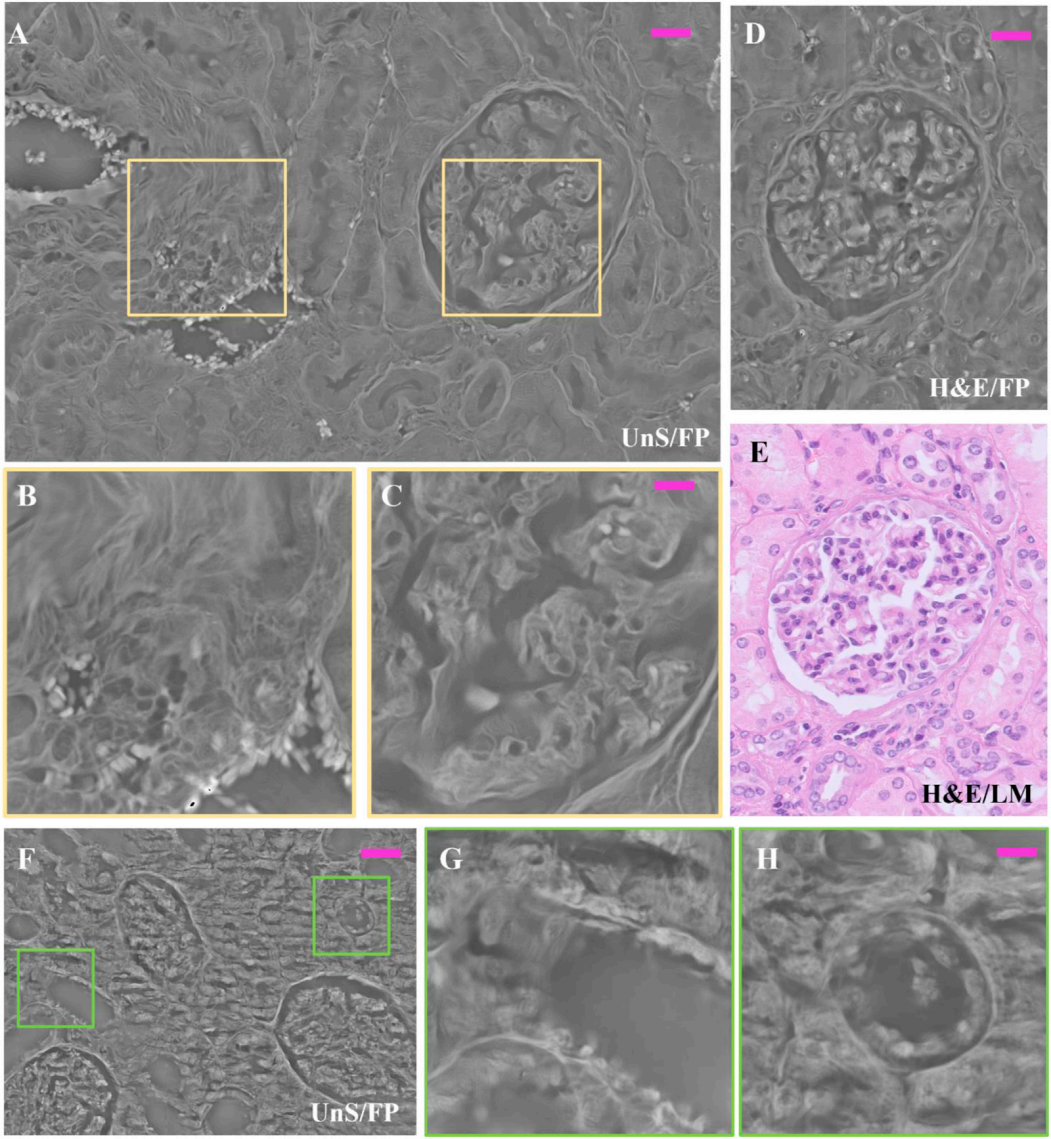
Unstained and H&E-stained renal tissue details: **(A)** Unstained renal tissue captured by FP, 3 µm thick, whose zoomed patches delimited by the purple boxes are respectively: **(B)** the smooth muscle tissue of a vasal section; and **(C)** the inner structure of the glomerulus; **(D)** same H&E-stained glomerulus captured by FP, 3 µm thick; **(E)** H&E-stained glomerulus captured by LM, 3 µm thick as gold standard; **(F)** different FP renal tissue slide, 3 µm thick and unstained; **(G,H)** renal tubule sections, zoomed in. The scale bar in **(A,D)** is 25 μm, while in **(B,C,G,H)** is 12 μm; in **(F)** is 50 µm.

Indeed, in Figure 5 the renal tissue portion shows how the morphology of both glomerulus and vasal sections is represented with tiny high-resolution details in the FP phase images of the 3 µm thick unstained slide (Figure 5A); detailed zooms of the area in A marked with colored boxes are shown in.

Figures 5B, C. In Figure 5B, the smooth muscle fibers of the vasal section are well-structured, and even the single red blood cells are well visible since they exhibit large phase-contrast. This is important evidence of the possibility of FP to map in one single image mm^2^-scale tissue areas while scaling down resolution up to the single cell level. The inner structure of the glomerulus is well represented in the zoom of Figures 5C, D shows a H&E-stained FP phase map, where a glomerulus is shown. This slightly differs from the unstained glomerulus of Figure 5A since the cell nuclei are more visible due to the presence of the H&E stain that changes the local light absorption properties of this tissue structure. The reference H&E-LM image is reported in Figure 5E for the sake of comparison. In Figures 5A–F different unstained area, 3 µm thick, is reported. The zooms in details of Figures 5G–H show how the architecture of the tubules sections appears in the FP phase map. Moreover, a 10 μm thick unstained renal tissue slide has been scanned by means of remote-controlled motorized stages to cover an area of 167.6 ${mm}^{2}$ , (17.1 × 9.8 mm). The final high-resolution FP image results from the combination of 63 FoVs, which still maintain a resolution of 0.5 μm. A portion of the previous scanned FP phase.

**FIGURE 6:**
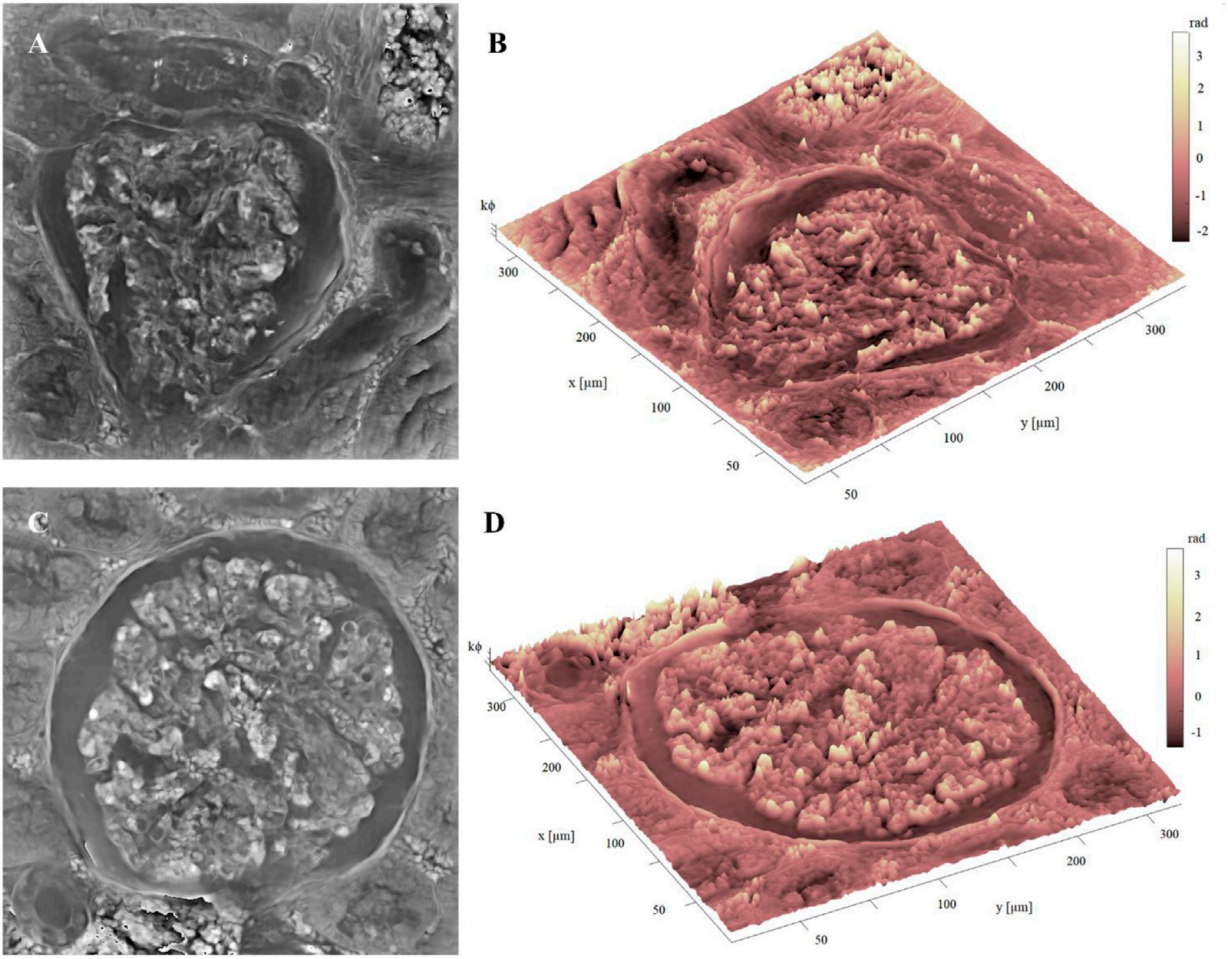
Unstained glomerulus details: **(A)** FP phase map of unstained glomerulus, captured by FP, 10 µm thick; **(B)** Pseudo3D plot of the glomerulus in **(A)**, whose axes represent the real dimensions of the samples; **(C)** FP phase map of a different unstained glomerulus, captured by FP, 10 µm thick; **(D)** pseudo3D plot of the glomerulus in **(C)**. The color-bar represents the phase values in radiants, multiplied by a k constant equal to 10 for the sake of better visualization.

**FIGURE 7:**
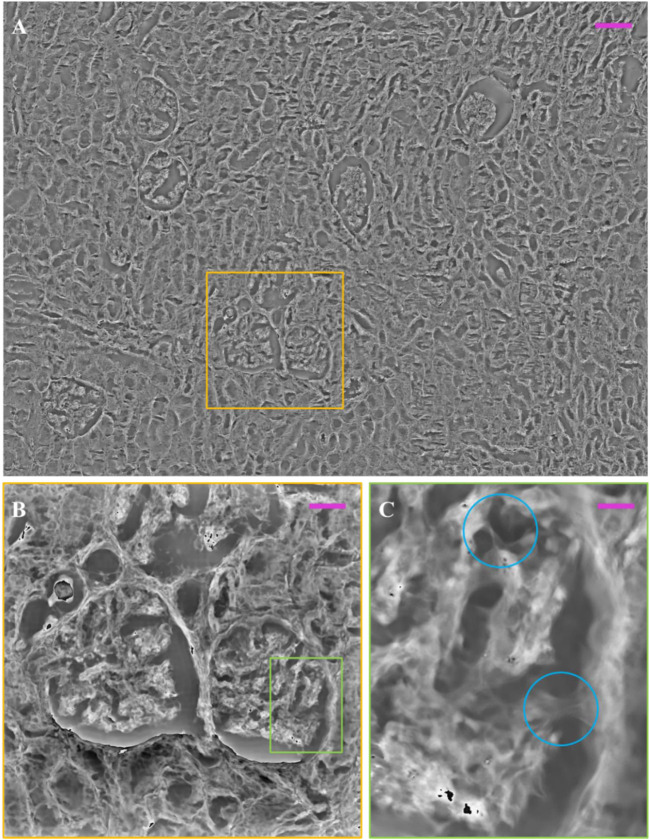
Multi-scaled/unstained FP phase image over our FP field of view: **(A)** FP phase map of unstained renal tissue, captured by FP, 10 µm thick over 3.3 mm^2^; **(B)** zoomed glomeruli and tubules sections (yellow box); **(C)** zoom of glomerulus inner structures (green box). The light blue circles highlight the basal membranes of the glomerulus. The scale bars are respectively: ∼105 ∼58, and ∼10 µm.

Image, (i.e., the stitching of 16 FoVs out of 63) is available in the repository link. Here we show two glomeruli obtained from this set of measurements. In Figures 6A–C, the FP phase maps show the fine details of glomeruli architecture, which are correctly exhibited with good contrast despite the histological slide was not stained. Comparing the 10 µm thick unstained glomerulus (see, e.g., Figure 6C, D and the 3 µm thick glomerulus of Figures 5A, C greater depth is perceived in the 10 µm glomerulus, highlighting the three-dimensionality in the internal structure. This is clearly apparent from the pseudo-3D plots of both glomeruli, Figures 6B–D, where the colormap indicates the phase levels. In Figure 7, an unstained kidney tissue slide, 10 µm thick, has been reported over the entire FoV that FP setup allows to obtain ( ∼ 3.3 mm^2^), Figure 7A. Comparing the unstained tissue slide 10 µm thick with the unstained one 3 µm thick in Figure 5, the difference in phase contrast due to the thickness of the sample is noted and the inner structures of glomeruli and tubules sections are still detailed as you can see in Figures 7B, C. The higher the phase values are, the more depth is perceived of the renal tissue without generating artifacts in the phase contrast image. Both in Figure 5C and in Figure 7C, the basal membranes of the glomerulus are visible, which is useful to understand, in future, the pathological states of the basal membranes without using stains.

**FIGURE 8:**
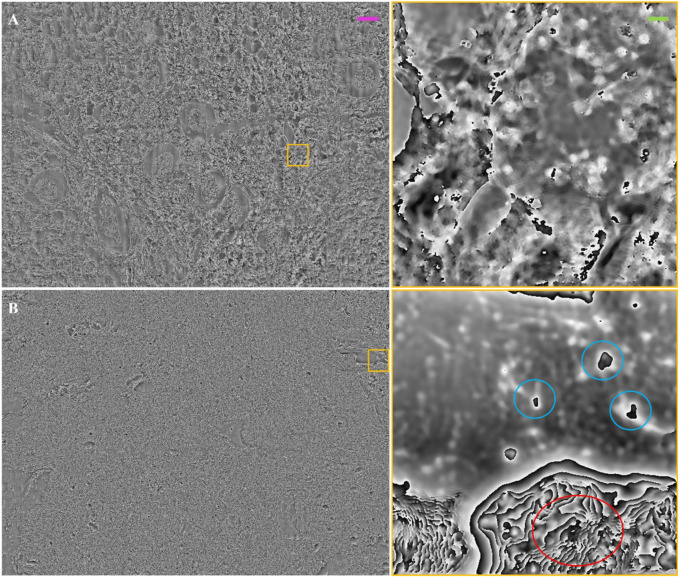
Thick/unstained FP phase images: **(A)** FP phase map of unstained renal tissue 20 µm thick. The inset on the right is a zoom of the yellow box; **(B)** FP phase map of unstained renal tissue 30 µm thick. The inset on the right is a zoom of the yellow box. The light blue circles show tissue elements that behave as pure phase objects, for which the thin object assumption stands, and after phase wrapping can return the optical thickness measure. The red circle is a wrapped phase zone where the phase might be not defined due to light scattering or absorption from the thick tissue. The purple scale bar for the full field of view images is ∼105 μm, while the green one on the zoomed patches is ∼7.5 µm.

Furthermore, we probed thicker renal tissue slides, 20 μm (Figure 8A) and 30 μm (Figure 8B) thick, to evaluate the limits of our FP system. For both images, phase jumps are present. In Figures 8A, B, the phase maps of the thicker renal slides present certain areas with elements that behave as pure phase objects, i.e., for which the thin sample assumption stands; while other areas of the slide, although the thickness is the same, present higher inhomogeneities, which makes the thin sample assumption not valid. In these cases, a different light propagation model and in turn a different FP reconstruction algorithm should be used, e.g., the multi-slice model under the first Born or Rytov approximations (Konda P. C. et al., 2020). For instance, if the light-blue circled areas were unwrapped (Figure 8B right-side), the quantitative information would be maintained. This might not happen in the areas highlighted by red circles, where such models to treat highly inhomogeneous specimens need to be employed to gather the quantitative phase-contrast image. In view of these observations, we conclude that such more complex phase-retrieval approaches have to be used to extend the FP observation of kidney tissue slides thicker than 10 µm.

In the repository link we reported an unstained FP phase contrast image of renal tissue slide 3 µm thick, the relative H&E stained one, and the LM counterpart, where it is possible to directly prove the high resolution over the wide field of view of the FP images.

## 3 Discussion and conclusion

Several microscopic techniques are currently employed for renal analysis (Angelotti et al., 2021), which are complementary to study kidney morphology and physiopathology. Here, we presented for the first time an alternative methodology to investigate renal tissue slides by a quantitative phase-contrast FP microscopy.

The FP images give the possibility to reach a gigapixel space-bandwidth product, overcoming the trade-off between high resolution and wide field of view, in a stain-free manner.

Imaging tissue slides without marking physiological and pathological regions of interest by staining characterizes the FP method with respect to the conventional microscopy techniques, allowing to avoid tissue alteration due to the staining and to carry out quantitative measurements from phase contrast values (Konda P. C. et al., 2020; Wakefield Devin et al., 2022). The staining free modality is helpful specially to image live cells without compromising cellular physiology, behavior and shape with invasive procedures due to dyes application, e.g., phototoxicity (Kasprowicz et al., 2017). The quantitative phase measurements (Cacace et al., 2020) allow extracting the sample optical thickness, which includes both physical thickness and refractive index variation Beuthan et al., 1996, as well the sample size measure and its biovolume (Merola et al. 2018). In case of cellular specimens, the dry mass can be estimated as important parameter of cell growth related to protein information (Barer R., 1952; Wang et al., 2021). Changes in the previous parameters can be physiological and pathological alerts associated with the sample. Moreover, quantitative features from FP images could be extracted to describe and characterize cells and tissues, adding a different kind of information to pathomic studies that could identify new diagnostic and prognostic biomarkers with a view to the context of personalized medicine (Fan et al., 2022). This quantitative information could also be combined with clinical/laboratory information and other omics data such as genomic and radiomic data in order to improve diagnostics and molecular knowledge about diseases (Saltz et al., 2017).

The main objective of this work is to show to physicians how kidney tissue structures appear in FP images, comparing them with the LM counterparts, considered the imaging gold standard in histology. In particular, it is noteworthy to underline the capability of FP phase maps to enhance renal components without using stains, Figures 4–7. Since the unstained FP phase contrast images are quantitative, important features can be measured, which can fully characterize renal tissue properties.

Of course, FP modality has some limitations when coupled to staining processes. For instance, we noticed that depending on the stain used, some slide portions can scatter light provoking phase ambiguities. In fact, looking at Figure 2C, in the zoomed patch of the CKpool-stained phase image (left-side), some phase jumps are visible since phase values are ranged in [-π, π]. This suggests that CKpool staining is not preferable to be coupled to FP analysis. We could stain kidney tissues with different antibodies using a different chromogen like e.g., Texas red in future experiments to evaluate their suitability with FP method. Nevertheless, the phase ambiguities can be resolved by applying unwrapping algorithms (Bioucas-Dias and Valadao, 2007), which can take out-of-range phase values, avoiding such phase jumps.

The optimum FP phase-retrieval method to employ depends on the thickness of the tissue slide and its inhomogeneities. In this work, tissue slides until 10 µm of thickness can be processed using the simpler Alternate Projection algorithm based on the weak scattering assumption. Conversely, thicker tissue slides, such as 20 and 30 µm thick, have shown phase-contrast images affected by phase jumps, Figure 8. For these cases, multi-slice light propagation models should be used to cope with large RI inhomogeneities in the 3D distribution of the slide.

Besides, measuring the morphometric features of 10 µm tissue slides in stain-free mode is a very promising result making FP a broadly exploitable imaging method in tissue histology. Indeed, the use of stains typically limits the maximum thickness of the slides analyzable by LM, as fluorescence emissions from the large tissue volumes makes hard to localize the source of each fluorescent signal in the 2D LM image. In general, very thick stained tissue slides, e.g., 10 μm thick, subject to H&E staining, are not useful for kidney physiology or pathology. Further studies will be devoted to probe tissue slides with thickness ranging between 10 and 20 µm and to use multi-slice light propagation models to extend the applicability of FP analysis of kidney tissues.

A further limit could be the computational burden associated with the FP reconstruction process, which requires to combine the Fourier spectra of multiple acquisitions and to solve an iterative phase retrieval process. However, this problem can be solved by using a deep learning approach, where a pretrained neural network is asked to generate FP reconstructions in real time (Bianco et al., 2022).

We propose FP method to be an alternate imaging tool that can find application in clinical practice due to the low-cost implementation and the possibility to be automatized for inexperienced operators. Additional investigations are necessary to validate FP to other types of tissue slides, both healthy and pathological, as well as to explore the potential of the method for further analysis on FP-based quantitative features that can be used to better characterize tissue samples and performing more reliable and accurate predictions of clinical outcomes. We are ongoing to examine kidney biopsies with different pathologies, such as membranous glomerulonephritis, proliferative glomerulonephritis, tubular lesions to prove the capability of FP to highlight in phase contrast images, retrieved in stain-free modality, the peculiarities of the different pathologies under FP analysis. A future perspective is the possibility, by means of FP, to discriminate healthy and sick renal tissue slides relying on features extraction from FP phase contrast images in combination with pathomic features to strengthen the reliability of the clinical outcome. The FP image analysis is aimed to help physicians in diagnostic evaluation.

## 4 Materials and methods

### 4.1 Sample preparation

Healthy tissues of two nephrectomy of benign lesions (oncocytoma and angiomyolipoma) were processed routinely with Logos Milestone microwave processor, cut at different thickness (3, 10, 20 and 30 µm) and 3 µm sections were stained in H&E with Sakura Tisse Tek Prisma Plus and IIC for CKpool; while FVIIIra, and AML were performed using Roche Ventana BenchMark according to

Routine protocols in use in the Lab, stained with DAB. Moreover, other 3 µm sections were stained with Blue Tol and Giemsa. Serial unstained sections of 3, 10, 20 and 30 µm were de-paraffined and mounted with Sakura Tisse Tek Prisma Plus as stained ones. All stained slides were scanned with Philips UFS scanner at real magnification of × 40. One 10 µm and few 3 µm unstained sections after FP methodology were stained with H&E to compare the same images taken from the same slide with the two different techniques.

### 4.2 Fourier ptychographic microscopy principle

Basically, the conventional microscopy images are realized by choosing either high lateral resolution or large field of views (FoVs). The microscope objectives with large numerical apertures (NAs) ensure high magnification and high resolution, but limiting the imaged FoV, while small magnification and small NAs typically provide wide FoVs while sacrificing the lateral resolution (Rottenfusser, 2013). FP microscopy overcomes this limitation by ensuring both super resolution and wide FoVs exploiting the principle of synthetic numerical aperture (Zheng et al., 2013). The low NA objective lens, used in the FP system, guarantees wide FoVs. In order to enhance the poor resolution allowed by the low NA microscope objective employed, the FP system is designed in order to illuminate the sample from multiple angles in a sequential mode, usually by means of a LED matrix. Multiple low-resolution images are captured accordingly. Then, the FP reconstruction process is devoted to furnish the synthesis of a bigger NA, combining the contribution of each inclined light wave in the Fourier domain and solving a phase-retrieval problem, i.e., the problem of estimating the phase of an object from a set of intensity measures.

Let consider *s(* ***r*** *)* as the tissue sample notation, displaced on the object plane, and $e^{j2\pi f_{i}r}$ as a simple formulation of the complex field generated by the i-th LED of the N dimensional source-matrix, where ***r*** refers to the system spatial coordinates ( $x_{i},y_{i}$ ), $f_{i}$ is the spatial frequency vector and ***N*** is the number of LEDs used for probing. Thus, when light passes through the tissue sample, the transmitted complex sample field can be expressed by:

$$t\left(r\right)={s\left(r\right)e}^{j2\pi f_{i}r}$$ (1)

**FIGURE 9:**
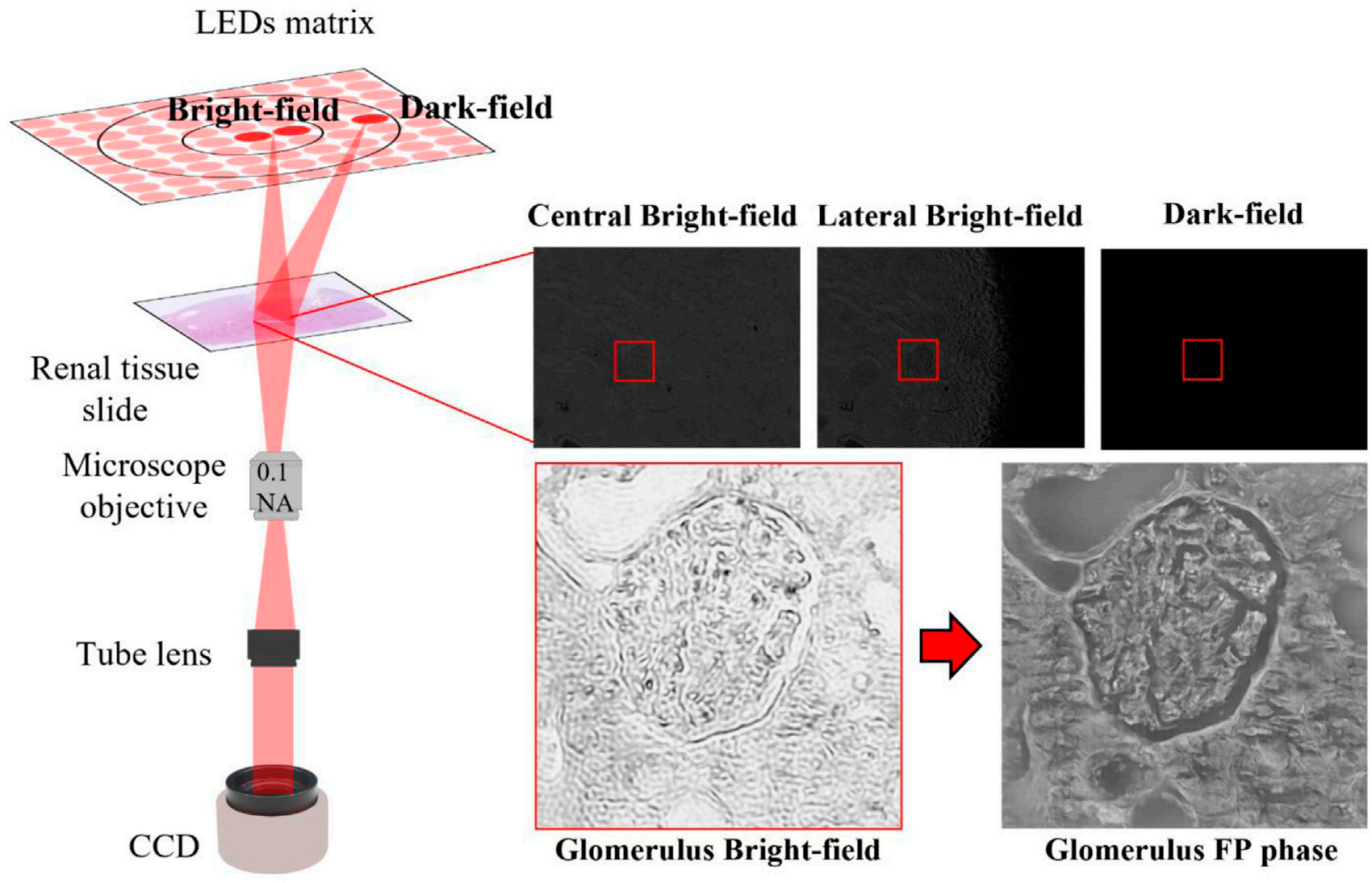
FP setup: Left side: sketch of FP setup; Right-side: raw images acquired according to the LED switched on. The glomerulus bright field after iterative phase retrieval algorithm appears highly resolved in amplitude and phase maps.

The spatial frequency vector strictly depends on the angle $\alpha_{i}$ that the i-th illumination wave forms with the optical axis of the FP system, in particular:

$$f_{i}=\left(f_{x_{i}},f_{y_{i}}\right)=-\frac{1}{\lambda }\left(\sin\alpha_{x_{i}},\sin\alpha_{y_{i}}\right)=-\frac{1}{\lambda }\left(\sin\left(\tan^{-1}\left(\frac{x_{i}}{d}\right)\right),\sin\left(\tan^{-1}\left(\frac{y_{i}}{d}\right)\right)\right)$$ (2)

where ***d*** is the distance between the LEDs matrix and the object plane, Figure 9.

Hence, the transmitted complex sample field convolves with the impulsive response $h\left(r\right)$ of the microscope objective before impacting the sensor device (camera). Here, the camera measures the low-resolution (LR) intensity of the tissue sample as follows:

$$I_{LR,i}={\left|t\left(r\right)\;\;*\;\;h\left(r\right)\right|}^{2}\;\overset{\mathrm{FT}}{\Rightarrow }\;I_{LR,i}={\left|{FT}^{-1}\left\{T\left(f-f_{i}\right)\;H\left(f\right)\right\}\right|}^{2}$$ (3)

Trivially, the convolution product among $t\left(r\right)$ and $h\left(r\right)$ is the inverse Fourier transform ( ${FT}^{-1}$ ) of a simple product between their Fourier transforms, $T\left(f\right)$ and $H\left(f\right)$ respectively. Noteworthy, $H\left(f\right)$ is the transfer function of the optical system, which depends on the microscope objective NA and the wavelength of the light source.

The central LEDs, due to smaller angles that correspond to the lowest spatial frequencies in the Fourier domain, generate the so-called bright field images, Figure 9; while the external LEDs, with more angled light waves, produce the dark-field images, which contain the tissue sample details since correspond to the highest spatial frequencies. Furthermore, each LED has its own pupil aperture in the Fourier spectrum in the corresponding spatial frequency coordinates, thus filtering only the sample information at those spatial frequencies. Properly stitching the spectral contribution of the captured images, a larger numerical aperture is synthesized as:

$${NA}_{syn}={NA}_{MO}+\underset{i}{\sum }{NA}_{i}$$ (4)

The equation addends are ${NA}_{MO}$ , i.e., the numerical aperture of the microscope objective lens and $\underset{i}{\sum }{NA}_{i}$ **,** i.e., the sum of LEDs pupil apertures in the Fourier domain. Eq. 4 clearly shows how the final resolution of the image improves as a result of the sum of illumination NAs to the conventional ${NA}_{MO}$ contribution.

The main goal of a quantitative phase imaging technique is to retrieve the phase information from the measurements. Nevertheless, the camera provides the sole intensity image of the object. Therefore, to get the complex object amplitude at high-resolution, phase retrieval algorithms can be applied (Yeh et al., 2015). These algorithms are iterated until convergence, passing from the Fourier domain to the space domain according to fixed constraints (Zheng et al., 2013). After converging, the high-resolution complex object field is available:

$$C\left({I_{LR}}^{\left(0\right)},f\right)={A\left(I_{LR},f\right)e}^{j\varphi \left({I_{LR}}^{\left(0\right)},f\right)}$$ (5)

where ${I_{LR}}^{\left(0\right)}$ , i.e., the initial guess, and f , i.e., the nominal spatial frequencies vector, are the constraint variables of the iterative algorithm. Hence, the amplitude, $A\left({I_{LR}}^{\left(0\right)},f\right)$ , and phase, $\varphi \left({I_{LR}}^{\left(0\right)},f\right)$ of the tissue samples are retrieved.

### 4.3 FPM experimental setup

Data acquisition has been performed using a custom-built FPM microscope, Figure 9. The angled illumination is provided by a matrix of light sources, where 32 x 32 RGB LEDs are surface-mounted. Each color channel, respectively at wavelengths of 632 nm (red), 532 nm (green) and 472 nm (blue), offers a spatially coherent quasi-monochromatic wave with a bandwidth of ∼20 nm. Kidney tissue samples, in our experiments, are probed with the 177 red LEDs closest to the center, describing a circular shape in a sequential mode. In particular, the LEDs are 4 mm apart. The LEDs illumination process is guided with an Arduino board in combination with Matlab^®^ scripts. Each LED angled wave impacts on the object plane, 4.67 cm away from the LEDs matrix. The tissue sample transmitted wave is collected by a × 4 plan achromatic objective lens (Plan N, 0.1 NA, Olympus) and then, passing through a 400 mm tube lens, arrives on a charge-coupled device (CCD) camera (Photometrics Evolve 512), with 4.54 μm pixel pitch. The low-resolution images, captured by the CCD camera with a 12-bit quantization, are characterized by a × 4.29 samples magnification and a square pixel size of 1460 × 1940.

To process the FPM images, the full FoV is divided into small patches sized 100 × 100, thus consisting of 266 patches. The phase retrieval algorithm is applied to each low-resolution patch, which finally will have a bigger dimension, i.e., 500 × 500 pixels. The high-resolution entire FoV comes out stitching the 266 resulted patches, reaching a size of 7000 × 9500 pixels. Our FPM system provides a resolution of 0.5 µm, on a ∼3 ${mm}^{2}$ FoV area. To remove the raster grid artifact, an overlapping of 40 pixels in the low-resolution image, which corresponds to an overlapping of 200 pixels in the high-resolution reconstructed image has been considered for the used stitching algorithm (Chalfoun, J. et al., 2020).

We ran the FPM algorithm on a machine mounting an Intel i7-4790 CPU running @3.60 GHz and 16 GB RAM. The computational time for a 100 × 100 patch is 7.2 s, iterating the FPM algorithm 60 times. The total number of the stitched patches is 266, employing ∼32 min for one entire slide. If we consider a large overlap between patches for accurately minimizing stitching artifacts, the total time for one slide grows to ∼86 min. In order to reduce the computational time while keeping the overlap between patches and minimizing stitching artifacts, two strategies can be adopted. The former is to use parallel processing approaches in multicore or multimachine implementations. Indeed, FPM reconstruction is a highly parallelizable algorithm and each patch can be processed independently. The latter is to use deep learning architectures, namely, Generative Adversarial Networks (GAN) previously trained to emulate the entire phase-retrieval process (Bianco et al., 2022).

## Data Availability

We provide a repository link (https://doi.org/10.5281/zenodo.7418567), where readers can visualize a FP phase contrast image of a 3 μm thick unstained renal tissue slide and the FP phase contrast image of the same region after staining with H&E. In the repository readers can compare the previous images with the corresponding H&E bright image captured by conventional microcopy of the same region on a different section. Moreover, we report a FP phase contrast image of a 10 μm thick unstained renal tissue slide, which has been obtained by stitching 16 FP fields of view to image a big portion of the kidney tissue slide under analysis.

## References

[B1] Angelotti M. L. Antonelli G. Conte C. Romagnani P. (2021). Imaging the kidney: From light to super-resolution microscopy. Nephrol. Dial. Transplant. 36 (1), 19–28. official publication of the European Dialysis and Transplant Association - European Renal Association. 10.1093/ndt/gfz136 31325314 PMC7771978

[B2] Azevedo Tosta T. A. de Faria P. R. Neves L. A. do Nascimento M. Z. (2019). Computational normalization of H&E-stained histological images: Progress, challenges and future potential. Artif. Intell. Med. 95, 118–132. 10.1016/j.artmed.2018.10.004 30420242

[B3] Barer R. (1952). Interference microscopy and mass determination. Nature 169, 366–367. 10.1038/169366b0 14919571

[B4] Beuthan J. Minet O. Helfmann J. Herrig M. Muller G. (1996). The spatial variation of the refractive index in biological cells. Phys. Med. Biol. 41, 369–382. 10.1088/0031-9155/41/3/002 8778819

[B5] Bianco V. Delli Priscoli M. Pirone D. Zanfardino G. Memmolo P. Bardozzo F. (2022). Deep learning-based, misalignment resilient, real-time fourier ptychographic microscopy reconstruction of biological tissue slides. IEEE J. Sel. Top. Quantum Electron. 28 (4), 1–10. Art no. 6800110. 10.1109/JSTQE.2022.3154236

[B6] Bianco V. Mandracchia B. Běhal J. Barone D. Memmolo P. Ferraro P. (2021). Miscalibration-tolerant fourier ptychography. IEEE J. Sel. Top. Quantum Electron. 27 (4), 1–17. 10.1109/JSTQE.2020.3025717

[B7] Bioucas-Dias J. M. Valadão G. (2007). Phase unwrapping via graph cuts. IEEE Trans. Image Process 16 (3), 698–709. 10.1109/tip.2006.888351 17357730

[B8] Cacace T. Bianco V. Ferraro P. (2020). Quantitative phase imaging trends in biomedical applications. Opt. Lasers Eng. 135, 106188–188. 10.1016/j.optlaseng.2020.106188

[B9] Chalfoun J. Majurski M. Blattner T. Bhadriraju K. Keyrouz W. Bajcsy P. (2020). Mist: Accurate and scalable microscopy image stitching tool with stage modeling and error minimization. Sci. Rep. 7, 4988. 10.1038/s41598-017-04567-y PMC5504007 28694478

[B10] Chandra K. P. Lars L. Zhou K. C. Xu S. Harvey A. R. Roarke H. (2020). Fourier ptychography: Current applications and future promises. Opt. Express 28, 9603–9630. 10.1364/OE.386168 32225565

[B11] Chlipala E. A. Butters M. Brous M. Fortin J. S. Archuletta R. Copeland K. (2021). Impact of preanalytical factors during histology processing on section suitability for digital image analysis. Toxicol. Pathol. 49 (4), 755–772. 10.1177/0192623320970534 33251977 PMC8091422

[B12] Chozinski T. J. Mao C. Halpern A. R. Pippin J. W. Shankland S. J. Alpers C. E. (2018). Volumetric, nanoscale optical imaging of mouse and human kidney via expansion microscopy. Sci. Rep. 8, 10396. 10.1038/s41598-018-28694-2 29991751 PMC6039510

[B13] Chung J. Ou X. Kulkarni R. P. Yang C. (2015). Counting white blood cells from a blood smear using fourier ptychographic microscopy. PLoS One 10 (7), e0133489. 10.1371/journal.pone.0133489 26186353 PMC4506059

[B14] Fan B. Huang Y. Zhang H. Chen T. Tao S. Wang X. (2022). Analysis of genetic profiling, pathomics signature, and prognostic features of primary lymphoepithelioma-like carcinoma of the renal pelvis. Mol. Oncol. 16, 3666–3688. 10.1002/1878-0261.13307 36052737 PMC9580896

[B15] Howell D. N. Herrera Guillermo A. (2021). Electron microscopy in renal pathology: Overall applications and guidelines for tissue, collection, preparation, and stains. Ultrastruct. Pathol. 45 (1), 1–18. 10.1080/01913123.2020.1854407 33320036

[B16] Hwang W.-J. Cheng S.-C. Cheng C.-J. (2011). Efficient phase unwrapping architecture for digital holographic microscopy. Sensors 11, 9160–9181. 10.3390/s111009160 22163688 PMC3231254

[B17] Judge T. R. Bryanston-Cross P. J. (1994). A review of phase unwrapping techniques in fringe analysis. Opt. Lasers Eng. 21 (4), 199–239. 10.1016/0143-8166(94)90073-6

[B18] Kasprowicz R. Suman R. O’Toole P. (2017). Characterising live cell behaviour: Traditional label-free and quantitative phase imaging approaches. Int. J. Biochem. Cell Biol. 84, 89–95. 10.1016/j.biocel.2017.01.004 28111333

[B19] Kim S. W. Roh J. Park C. S. (2016). Immunohistochemistry for pathologists: Protocols, pitfalls, and tips. J. Pathol. Transl. Med. 50 (6), 411–418. Epub 2016 Oct 13. PMID: 27809448. 10.4132/jptm.2016.08.08 27809448 PMC5122731

[B44] Konda P. C. Lars L. Zhou K. C. Xu S. Harvey A. R. Horstmeyer R. (2020). Fourier ptychography: Current applications and future promises. Opt. Express 28, 9603–9630. 10.1364/OE386168 32225565

[B20] Merola F. Memmolo P. Miccio L. Mugnano M. Ferraro P. (2018). Phase contrast tomography at lab on chip scale by digital holography. Methods 136, 108–115. 10.1016/J.YMETH.2018.01.003 29341925

[B31] Montrésor S. Memmolo P. Bianco V. Ferraro P. Picart P. (2019). Comparative study of multi-look processing for phase map de-noising in digital Fresnel holographic interferometry. J. Opt. Soc. Am. A 36 (2), A59–A66. 10.1364/JOSAA.36.000A59 30874091

[B21] Ou X. Horstmeyer R. Yang C. Zheng G. (2013). Quantitative phase imaging via Fourier ptychographic microscopy. Opt. Lett. 38, 4845–4848. 10.1364/OL.38.004845 24322147 PMC4277232

[B22] Park S. Kim Y. Moon I. (2021). Automated phase unwrapping in digital holography with deep learning. Biomed. Opt. Express 12, 7064–7081. 10.1364/BOE.440338 34858700 PMC8606148

[B23] Park Y. Depeursinge C. Popescu G. (2018). Quantitative phase imaging in biomedicine. Nat. Phot. 12, 578–589. 10.1038/s41566-018-0253-x

[B24] Pirone D. Bianco V. Valentino M. Mugnano M. Pagliarulo V. Memmolo P. (2022). Fourier ptychographic microscope allows multi-scale monitoring of cells layout onto micropatterned substrates. Opt. Lasers Eng. 156, 107103. 10.1016/j.optlaseng.2022.107103

[B25] Popescu G. (2011). Quantitative phase imaging of cells and tissues. New York, NY, USA: McGraw-Hill Professional.

[B26] Rick R. Dörge A. Beck F. X. Thurau K. Dorge A. (1986). Electron-probe X ray microanalysis of transepithelial ion transport. Ann. N. Y. Acad. Sci. 483, 245–259. 10.1111/j.1749-6632.1986.tb34528.x 3494414

[B27] Rottenfusser R. (2013). Chapter 3 - proper alignment of the microscope. Methods Cell Biol. 114, 43–67. 10.1016/B978-0-12-407761-4.00003-8 23931502

[B28] Saltz J. Almeida J. Gao Y. Sharma A. Bremer E. DiPrima T. (2017). Towards generation, management, and exploration of combined radiomics and pathomics datasets for cancer research. AMIA Jt. Summits Transl. Sci. Proc. 2017, 85–94. 28815113 PMC5543366

[B29] Schuh C. D. Haenni D. Craigie E. Ziegler U. Weber B. Devuyst O. (2016). Long wavelength multiphoton excitation is advantageous for intravital kidney imaging. Kidney Int. 89 (3), 712–719. 10.1038/ki.2015.323 26509590

[B30] Shroff U. N. Schiessl I. M. Gyarmati G. Riquier-Brison A. Peti-Peterdi J. (2019). Novel fluorescence techniques to quantitate renal cell biology. Methods Cell Biol. 154, 85–107. 10.1016/bs.mcb.2019.04.013 31493823 PMC6748388

[B32] Song S. Kim J. Hur S. Song J. Joo C. (2019). Large-area, high-resolution birefringence imaging with polarization-sensitive fourier ptychographic microscopy. ACS Photonics 8 (1), 158–165. 10.1021/acsphotonics.0c01695

[B33] Su Q. Hu F. Liu Y. Ge X. Mei C. Yu S. (2018). Cryo-EM structure of the polycystic kidney disease-like channel PKD2L1. Nat. Commun. 9, 1192. 10.1038/s41467-018-03606-0 29567962 PMC5864754

[B34] Tian L. Liu Z. Yeh L-H. Chen M. Zhong J. Waller L. (2015). Computational illumination for high-speed *in vitro* Fourier ptychographic microscopy. Optica 2, 904–911. 10.1364/OPTICA.2.000904

[B35] Tsutsumi Y. (2022). Pitfalls and caveats in applying chromogenic immunostaining to histopathological diagnosis. Cells 10 (6), 1501. 10.3390/cells10061501 PMC8232789 34203756

[B36] Unnersjö-Jess D. Scott L. Blom H. Brismar H. (2016). Super-resolution stimulated emission depletion imaging of slit diaphragm proteins in optically cleared kidney tissue. Kidney Int. 89 (1), 243–247. 10.1038/ki.2015.308 26444032

[B37] Unnersjö-Jess D. Scott L. Zambrano Sevilla S. Patrakka J. Blom H. Brismar H. (2018). Confocal super-resolution imaging of the glomerular filtration barrier enabled by tissue expansion. Kidney Int. 93 (4), 1008–1013. 10.1016/j.kint.2017.09.019 29241621

[B38] Wakefield Devin L. Richard G. Wong K. Wang S. Hale C. Chung-Chieh Y. (2022). Cellular analysis using label-free parallel array microscopy with Fourier ptychography. Biomed. Opt. Express 13, 1312–1327. 10.1364/BOE.451128 35415005 PMC8973186

[B39] Wang Z. Bianco V. Pirone D. Memmolo P. Villone M. M. Maffettone P. L. (2021). Dehydration of plant cells shoves nuclei rotation allowing for 3D phase-contrast tomography. Light Sci. Appl. 10 (1), 187. 10.1038/s41377-021-00626-2 34526484 PMC8443563

[B40] Weening J. J. Jennette J. C. (2012). Historical milestones in renal pathology. Virchows Arch. 461 (1), 3–11. 10.1007/s00428-012-1254-7 22661128 PMC3400763

[B41] Williams Anthony J. Chung J. Ou X. Zheng G. Rawal S. Zheng A. (2014). Fourier ptychographic microscopy for filtration-based circulating tumor cell enumeration and analysis. J. Biomed. Opt. 19 (6), 066007. 10.1117/1.JBO.19.6.066007 24949708 PMC4572097

[B42] Yeh L-H. Dong J. Zhong J. Tian L. Chen M. Tang G. (2015). Experimental robustness of Fourier ptychography phase retrieval algorithms. Opt. Express 23, 33214–33240. 10.1364/OE.23.033214 26831989

[B43] Zheng G. Horstmeyer R. Yang C. (2013). Wide-field, high-resolution Fourier ptychographic microscopy. Nat. Phot. 7, 739–745. 10.1038/nphoton.2013.187 PMC4169052 25243016

